# miRNA-dependent resistance mechanisms to anti-hormonal therapies in estrogen receptor-positive breast cancer patients

**DOI:** 10.1016/j.omton.2025.200941

**Published:** 2025-01-28

**Authors:** Zainab Salam Al Hashami, Bert van der Vegt, Marian J.E. Mourits, Joost Kluiver, Anke van den Berg

**Affiliations:** 1 Department of Pathology and Medical Biology, University of Groningen, Groningen, the Netherlands; 2 University Medical Centre Groningen, Groningen, the Netherlands; 3 Department of Biochemistry, College of Medicine and Health Sciences, Sultan Qaboos University, Muscat, Oman; 4 Department of Gynaecological Oncology, University of Groningen, University Medical Centre Groningen, Groningen, the Netherlands

**Keywords:** MT: Regular Issue, estrogen receptor-positive breast cancer, anti-hormonal drug resistance, microRNAs, resistance mechanisms

## Abstract

The estrogen receptor (ERα) is expressed in 70%–80% of breast cancers and is a target of endocrine therapy. However, resistance to endocrine therapy poses a significant clinical challenge. MicroRNAs (miRNAs) have emerged as critical players in oncogenesis and as modulators of therapy response. This review provides an overview of miRNAs that modulate anti-hormonal drug responses. We identified 56 miRNAs associated with resistance to endocrine therapy. These miRNAs had a total of 40 proven target genes that were grouped based on their function under currently known resistance mechanisms, including ER modulation, signaling pathway activation, cell-cycle modulation, and other mechanisms. For a limited number of miRNA-target gene interactions, the relevance of the identified target gene(s) was confirmed by copy or rescue of the miRNA-induced phenotype. Overall, this review highlights critical roles of miRNAs as crucial mediators of resistance to anti-hormonal therapy. The identified miRNA-target gene interactions can serve as a foundation for future functional studies exploring the potential of selected miRNAs in overcoming drug resistance, which might improve outcomes for breast cancer patients.

## Graphical abstract

**Figure:**
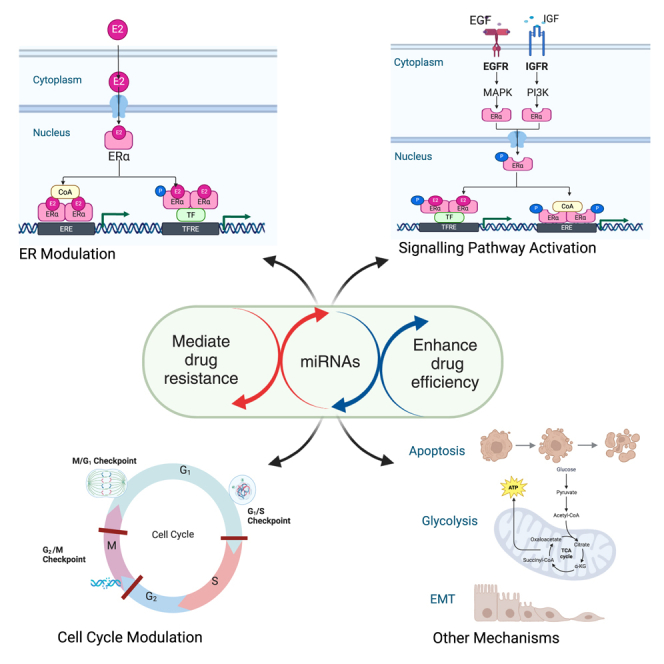

## Teaser

Al Hashami and colleagues provide an in-depth evaluation of miRNAs implicated in resistance to endocrine therapies in Erα^+^ breast cancer. By identifying 56 miRNAs and 40 associated target genes, this review provides an overview of miRNAs with critical regulatory roles within established resistance mechanisms and pinpoints potential novel therapeutic avenues.

## Introduction

Breast cancer is the most frequently diagnosed cancer in females worldwide, with 2.26 million new cases in 2020.^1^ It is the main cause of cancer-related deaths among women, accounting for 15.5% of all cancer-related deaths.^1^ Breast cancer is a complex, heterogeneous disease with a wide range of histological and molecular subtypes with variable clinical outcomes. The most prevalent subtype is estrogen receptor alpha-positive (ERα^+^ breast cancer, which accounts for 70%–80% of all cases.^2^

ERα is a member of the nuclear receptor superfamily that acts as a transcription factor to mediate the effect of estrogen.^3^ Upon binding to estrogen, ERα enters the nucleus of the cell and attaches to estrogen response elements, regulating the transcription of genes involved in cell growth, differentiation, and apoptosis.^4^ ERα triggering results in the activation of four distinct pathways: classical genomic pathway, nonclassical genomic pathway, estrogen-independent pathway, and nongenomic pathway.^5^ The classical and nonclassical genomic pathways involve binding of estrogen to ERα, which is followed by ERα dimerization, conformational change, and nuclear translocation. The classical genomic pathway involves a direct binding of ERα to specific sequences in the genome to regulate transcription of its target genes. The nonclassical genomic pathway depends on interactions of ERα with other transcription factors. The estrogen independent pathway involves phosphorylation of ERα by the overexpression of various factors, including epidermal growth factor receptor (EGFR) and Erb-B2 receptor tyrosine kinase 2 (ERBB2 or HER2).^6^ Stimulation of these factors triggers a multitude of signaling pathways like Ras/mitogen-activated protein kinase (MAPK), phosphoinositide 3-kinase (PI3K)/alpha serine/threonine-protein kinase (AKT)/mechanistic target of rapamycin kinase (mTOR), all pivotal in governing cell proliferation.^6^ In the nongenomic pathway, ERα dimers located close to the plasma membrane interact with protein kinases like Src and PI3K, which results in the activation of various protein-kinase cascades independent of gene transcription.^5^ ERα36 and ERα66 are two main isoforms of the ERα protein. ERα66 is the full-length receptor, while ERα36 is a smaller version lacking the N-terminal transactivation region. ERα66 acts through the two genomic pathways after translocating to the nucleus. ERα36 is located at the plasma membrane and plays a role in the non-genomic pathway. Both isoforms can modulate the effect of estrogen on different physiological processes.^5^^,^^7^

In breast cancer, activation of ERα drives tumor growth, making it a key target for hormone therapies such as tamoxifen and aromatase inhibitors (AIs).^3^^,^^4^ ERα^+^ breast cancer can be effectively treated with ER-targeting drugs. Tamoxifen, fulvestrant, and AIs are endocrine drugs that are widely used to treat ERα^+^ breast cancer patients. Tamoxifen is a selective estrogen receptor modulator that blocks the binding of estrogen to ERα and thereby decreases the transcriptional activity of Erα, resulting in reduced cell growth and enhanced cell death.^8^ Since its approval by the US Food and Drug Administration in 1977, tamoxifen has been the most widely prescribed anti-neoplastic drug worldwide. Fulvestrant is a selective estrogen receptor degrader that induces a conformational change of ERα, thereby accelerating its degradation. This leads to a long-term blockage of estrogen signaling and decreased progression.^9^ AIs decrease estrogen production by blocking aromatase, which is required to convert androgen to estrogen. AIs are used especially in postmenopausal women.^10^ All these drugs block estrogen-dependent activation of the estrogen receptor, making them effective approaches for the treatment of ER^+^ breast cancer patients.

Although highly effective, a substantial proportion of patients develop resistance to endocrine drugs. Currently known key endocrine resistance mechanisms include ER modulation, signaling pathway activation, cell-cycle modulation, stress signaling, and mechanisms related to the tumor microenvironment, nutritional stress, and metabolic regulation.^11^

Recent studies have shown that miRNAs are important players in the development of drug resistance in various cancers.^12^ miRNAs are short non-coding RNA molecules, which regulate gene expression by binding to mRNA and preventing their translation. Biogenesis of miRNAs starts with transcription of its host gene, also known as the primary miRNA, by RNA polymerase II. These transcripts are processed by the microprocessor complex consisting of at least Drosha and DiGeorge syndrome critical region gene 8 into precursor miRNAs. The precursors are transported from the nucleus to the cytoplasm by Exportin 5, where Dicer cuts them into small RNA duplexes. In general, one strand of the duplex is incorporated into the RNA-induced silencing complex, guiding it to target mRNAs based on limited sequence homology.^13^^,^^14^ They mostly bind to the 3′ UTR of their target mRNAs and repress protein production by destabilizing the mRNA molecule and translational silencing. A large proportion of human genes are under the control of miRNAs, resulting in a miRNA-dependent regulation of many crucial cellular processes, including cell differentiation, survival, proliferation, and cell death.^15^^,^^16^ Unraveling the role of miRNAs in cancer is complex due to their ability to target multiple genes within the same or different pathways. Numerous studies have shown that miRNAs play a role in all hallmarks of cancer.^17^^,^^18^ Based on this knowledge, miRNAs have been explored as potential therapeutic targets, with other potential therapeutic values being reported for miR-21, miR-10b, miR-155, and let-7 family members in breast, lung, pancreatic, and ovarian cancers.^18^^,^^19^ Several studies have shown that miRNAs play a role as biomarkers or in the pathogenesis of breast cancer.^20^^,^^21^ For example, the overexpression of miR-21 has been associated with advanced clinical stage, lymph node metastasis, and poor prognosis in breast cancer.^22^ Low miR-195 levels discriminated HER2^+^ from other breast cancer subtypes, while miR-210 and miR-148a were associated with relapse-free survival in breast cancer.^23^^,^^24^

**Figure 1:**
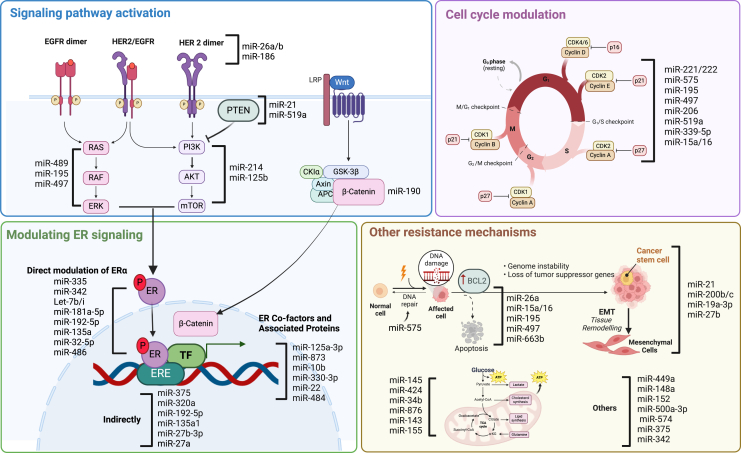
miRNA-mediated anti-hormonal drug resistance miRNAs are grouped based on their function under currently known resistance mechanisms, including ER signaling modulation, signaling pathway activation, cell-cycle modulation, and others (created with BioRender.com).

In this review, we aimed to provide an overview of the role of miRNAs in the development of endocrine drug resistance in ER^+^ breast cancer and thus provide insights into the potential of novel miRNA-based treatments to overcome this resistance. Table 1 summarizes the findings of the selected manuscripts, including miRNAs modulating the effectiveness of endocrine therapy and their experimentally confirmed targets. Most studies started with a selection of potentially interesting miRNAs using a profiling approach focused on defining miRNAs differentially expressed between sensitive and resistant cell line models and/or patient samples. Subsequently, functional studies were done to identify relevant target genes and pathways explaining the mechanism of resistance. miRNA-target gene interactions linked to endocrine therapy resistance were linked to a broad spectrum of target genes and cellular processes. We grouped our findings under four currently known major resistance mechanisms: ER modulation, signaling pathway activation, cell-cycle modulation, and others (Figure 1).^11^

**Table 1:** miRNAs regulating anti-hormonal drug resistance in breast cancer

miRNA	Expression and effect^a^	Treatment	Target genes	Reference
**Modulating ER signaling**				

miR-335	↓R	tamoxifen	ESR1	Martin et al.^25^
miR-342	↓R	tamoxifen	ESR1	He et al.^26^ and Young et al.^27^
Let-7b/i	↓R	tamoxifen	ESR1	Zhao et al.^28^
miR-181a-5p	↑R	tamoxifen	ESR1	Andreeva et al.^29^ and Benedetti et al.^30^
miR-192-5p	↑R	tamoxifen	ESR1	Kim et al.^31^
miR-135a	↓R	tamoxifen	ESR1	Zhang et al.^32^
miR-27a	↓R	tamoxifen	ZBTB10	Ljepoja et al.^33^ and Li et al.^34^
miR-32-5p	↓R	tamoxifen	ESR1	Wang et al.^35^
miR-125a-3p	↓R	tamoxifen	CDK3	Zheng et al.^36^
miR-873	↓R	tamoxifen	CDK3	Cui et al.^37^ and Zhang et al.^38^
miR-10b	↑R	tamoxifen	HDAC4	Ahmad et al.^39^
miR-330-3p	↑R	tamoxifen	HDAC4	Zhang et al.^40^
miR-22	↑R	fulvestrant	HDAC4, FOXP1	Wang et al.^41^
miR-320a	↓R	tamoxifen	ESRRA, PPP1R1B	Lü et al.^42^
miR-192-5p	↑R	tamoxifen	ESRRA	Kim et al.^31^
miR-135a1	↓R	tamoxifen	ESRRA, NCOA1	Zhang et al.^32^
miR-27b-3p	↓R	tamoxifen	NR5A2, CREB1	Zhu et al.^43^
miR-484	↓R	tamoxifen	KLF4	Wei et al.^44^
miR-375	↓R	tamoxifen	MTDH	Fu et al.^45^ and Ward et al.^46^
miR-486-5p	↓R	tamoxifen	HMGA1	Mansoori et al.^47^

**Activation of signaling pathways**				

miR-26a/b	↓R	tamoxifen	ERBB2	Tan et al.^48^
miR-186-3p	↓R	tamoxifen	EREG	He et al.^49^
miR-21	↑S	tamoxifen, fulvestrant	PTEN	Yu et al.^50^
miR-489	↓R	tamoxifen	MAPK14, PTPN11	Soni et al.^51^
miR-214	↓S	tamoxifen, fulvestrant	UCP2	Yu et al.^52^
miR-195, miR-497	↓R	tamoxifen	MAP2K1, RAF1, AKT3	Tian et al.^53^
miR-519a	↑R	tamoxifen	PTEN	Ward et al.^54^
miR-190	↑S	tamoxifen	SOX9	Yu et al.^55^
miR-155	↑R	tamoxifen	SOCS6	Shen et al.^56^
miR-125b	↑R	aromatase inhibitors	GSK3β, p70S6K	Vilquin et al.^57^

**Cell-cycle modulation**				

miR-221/222	↑R	tamoxifen	CDKN1B	Miller et al.^58^ and Wei et al.^59^
miR-575	↑R	tamoxifen	CDKN1B	Liu et al.^60^
miR-195, miR-497	↓R	tamoxifen	CCND1	Tian et al.^53^
miR-206	↓R	tamoxifen	WBP2	Tian et al.^61^
miR-519a	↑R	tamoxifen	CDKN1A, RB1	Ward et al.^54^
miR-339-5p	↓R	tamoxifen	CDK2	Feng et al.^62^
miR-15a/16	↓R	tamoxifen	CCNE	Chu et al.^63^

**Other mechanisms**				

miR-26a	↓R	tamoxifen	E2F7	Liu et al.^64^
miR-15a/16	↓R	tamoxifen	BCL2	Cittelly et al.^65^
miR-195, miR-497	↓R	tamoxifen	BCL2	Tian et al.^53^
miR-375	↓R	tamoxifen	HOX3B	Fu et al.^45^ and Ward et al.^46^
miR-200b/c	↓R	tamoxifen	MYB	Gao et al.^66^
miR-19a-3p	↑S	letrozole	CYP19A1	Xiang et al.^67^
miR-27b	↓R	tamoxifen	HMGB3	Li et al.^68^
miR-500a-3p	↓R	tamoxifen	LY6K	Kim et al.^31^
miR-449a	↓R	tamoxifen	ADAM22	Li et al.^69^
miR-148a/miR-152	↓R	tamoxifen	ALCAM	Chen et al.^70^
miR-574	↓S	tamoxifen	CLTC	Ujihira et al.^71^
miR-145-5p, miR-424-5p	↓R	tamoxifen	PSAT1	Petri et al.^72^
miR-34b-5p, miR-876-5p	↓R	tamoxifen	PHGDH	Petri et al.^72^
miR-143	↓R	aromatase inhibitors	HK2	Bacci et al.^73^
miR-575	↑R	tamoxifen	BRCA1	Liu et al.^60^
miR-663b	↑R	tamoxifen	TP73	Jiang et al.^74^
miR-342	↓R	tamoxifen	GEMIN4, BMP-7	Cittelly et al.^75^

a Down arrow (↓) represents decreased expression and up arrow (↑) represents increased expression of the miRNA; S indicates change of expression sensitizes breast cancer cells to endocrine therapy and R induces resistance in breast cancer cells.^11^

## ER modulation

The endocrine resistance mechanism grouped under ER modulation includes various processes that modulate the functionality of ERα. These mechanisms include loss of ERα combined with additional alterations to facilitate estrogen-independent growth; mutations in and fusions of ESR1; and binding of ERα to co-activators, co-repressors, transcription factors, nuclear receptors, and epigenetic modulators to enhance transcription of cell-cycle inducers.^11^ Of the 56 miRNAs identified as critical for defining the response to anti-hormonal therapy, 20 miRNAs target genes that fall within the group of ERα modulation. Besides targeting ERα or its isoforms, we identified 12 target genes that modulate ERα functionality and contribute to endocrine resistance.

Multiple miRNAs associated with sensitivity to endocrine therapy were shown to regulate expression of the estrogen receptor 1 (ESR1) encoding for ERα. Ectopic expression of these miRNAs, including miR-335, miR-342, let-7 family members, miR-181a, miR-192-5p, and miR-135a, resulted in decreased expression of ERα and reduced sensitivity of breast cancer cells to tamoxifen.^25^^,^^26^^,^^28^^,^^29^^,^^30^^,^^31^^,^^32^ However, in most of these studies, the relevance of the decrease in ERα in relation to the observed tamoxifen resistance has not been established. Additional data to support their relevance in drug resistance has been provided for part of the miRNAs targeting ERα. A critical role of miR-342 in response to tamoxifen was obtained by showing an association between high miR-342 levels and better overall and disease-free survival in gene expression data from The Cancer Genome Atlas program and from two Gene Expression Omnibus patient cohorts.^27^ Overexpression of let-7b, and to a lesser extent let-7a and let-7i, restored tamoxifen sensitivity in tamoxifen-resistant cells supporting the relevance of these miRNAs.^28^ Tamoxifen resistance was induced by prolonged suppression of ESR1 by the overexpression of miR-181a. The resistance was achieved via activation of PI3K/Akt signaling.^29^ Relevance of miR-135a was supported by a reduced level of miR-135a in tamoxifen-resistant cells and the observation that loss of the MIR135a1 locus or low miR-135a expression predicted resistance to endocrine therapies.^32^ Although all these studies confirmed targeting of ESR1 by each of the above-mentioned miRNAs, a direct mechanistic link between decreased ERα expression and the observed resistance has not always been indicated.

In contrast to the above-mentioned miRNAs, a decrease in the levels of miR-27a was found in tamoxifen-resistant cells, and this was associated with decreased levels of ERα. The overexpression of miR-27a was shown to increase the levels of ERα, while the inhibition of ERα led to a decrease in miR-27a, indicating a positive feedback loop. Moreover, breast cancer cells could be re-sensitized to tamoxifen by upregulating miR-27a.^33^ The underlying mechanism might involve direct targeting of zinc finger and BTB domain containing 10 (ZBTB10) by miR-27a.^34^ Both inhibition of miR-27a and overexpression of the miR-27a target gene ZBTB10 resulted in decreased ERα mRNA and protein levels in breast cancer.

miR-32-5p affected sensitivity to tamoxifen by specifically regulating expression of the shorter isoform of ESR1, ERα36.^35^ Mechanistically, this was shown to be dependent on cullin 4B (CUL4B). CUL4B represses transcription of miR-32-5p via epigenetic changes, resulting in increased levels of the miR-32-5p target ERα36 without affecting the levels of ERα66. Other miRNAs shown to modulate expression of ERα36 are members of the let-7 family.^28^ Strikingly, members of this miRNA family were also reported to target the full-length ESR1 transcript (see above), which has a completely different 3′ UTR as the ESR1 transcript coding for the ERα36 protein. Targeting efficiency of the let-7 family members to the 3′ UTR of the transcript encoding for ERα36 was most pronounced for let-7b and let-7i. The protein level of ERα36 was strongly increased upon inhibition of these miRNAs and decreased upon infection with let-7 mimics. Overexpression of the transcript coding for ERα36 lacking the 3′ UTR region with the let-7 binding sites restored the resistant phenotype of cells transfected with let-7.^28^ Thus, the interaction of these miRNAs with ERα36 was shown to be causal for the resistant phenotype.

Two miRNAs were shown to contribute to tamoxifen sensitivity via targeting cyclin-dependent kinase 3 (CDK3), which plays a pivotal role in the phosphorylation of ERα. Direct phosphorylation of ERα is an alternative way to activate the ER-signaling pathway, independent of its interaction with estrogen. Downregulation of miR-125a induced tamoxifen resistance by increased expression of its target CDK3.^36^ A second miRNA involved in tamoxifen resistance by targeting CDK3 is miR-873.^37^^,^^38^ Norcantharidin, a natural drug often used in China, was shown to induce the expression of miR-873, and this re-sensitized resistant cells to tamoxifen.^37^^,^^38^

Overexpression of miR-10b or miR-330-3p induced tamoxifen resistance in breast cancer cells by targeting histone deacetylase 4 (HDAC4).^39^^,^^40^ HDAC4 binds to the N terminus of ERα in the nucleus and regulates transcription of endogenous estrogen-responsive genes by recruitment of HDAC4 to the gene locus.^76^^,^^77^ Both increased and decreased miR-22 expression was shown to re-sensitize fulvestrant-resistant breast cancer cells to fulvestrant.^41^ Forced expression of miR-22 resulted in the downregulation of forkhead box P1 (FOXP1) and HDAC4, and a significant increase in the acetylation of histones targeted by HDAC4.^41^ FOXP1 has been reported to enhance estrogen-driven transcription.^41^

Decreased levels of miR-320a were seen in breast cancer samples and in tamoxifen-resistant cells. This reduction correlated with elevated expression of its target genes, estrogen-related receptor alpha (ESRRA) and protein phosphatase 1 regulatory inhibitor subunit 1B (PPP1R1B).^42^ Re-expression of miR-320a restored sensitivity to tamoxifen. This effect on tamoxifen sensitivity was shown to be modulated by targeting PPP1R1B and ESRRA, as well as their downstream targets Myc and CNND1.^42^ PPP1R1B and ESRRA are key regulators of cell proliferation, differentiation, and survival. PPP1R1B is a bifunctional signal transduction molecule that can act either as a kinase or a phosphatase inhibitor.^78^ Besides being targeted by miR-320a, ESRRA was targeted by miR-135a and miR-192-5p.^31^^,^^32^ ESRRA has a high homology with ESR1, but most likely does not bind to estrogen, and its ligand remains unknown.^79^ Previous work suggested that there may be overlap in the genes that are regulated by ERα and ESRRA^80^ and that ESRRA may modulate the activity of ERα.^81^ Enforced expression of miR-135a was also shown to target nuclear receptor coactivator 1 (NCOA1), and downregulation of both ESSRA and NCOA1 resulted in the inhibition of ERα signaling.^32^ NCOA1 is a transcriptional coactivator for steroid and hormone receptors, also known as RIP160 and SRC1. It was reported to play a role in ERα-mediated gene expression.^82^

Both *in vitro* and *in vivo* studies showed a critical role for miR-27b-3p, a second member of the miR-27 seed family, in endocrine therapy response. Downregulation of this miRNA induced tamoxifen resistance in breast cancer. The effect was linked to direct targeting of nuclear receptor subfamily 5 group A member 2 (NR5A2) and cyclic AMP (cAMP)-response element binding protein 1 (CREB1).^43^ Restoration of tamoxifen sensitivity was successfully achieved through the overexpression of miR-27b or by inhibiting NR5A2 and CREB1 in resistant cells, confirming their relevance.^43^ Both NR5A2 and CREB1 have known relationships with ERα. NR5A2 enhances recruitment of ERα to estrogen-responsive elements and thereby stimulates transcription of estrogen-responsive genes.^83^ In line with this finding, others showed that NR5A2 deletion attenuated ERα signaling in hepatocytes.^84^ Activation of CREB1 is induced by its phosphorylation in response to cAMP-activated protein kinase A. In the absence of estrogen, ERα can also be activated by cAMP signaling, which leads to re-direction of ERα to other transcription start sites, including MYC target genes.^85^

Downregulation of miR-484 was observed in cells exhibiting tamoxifen resistance. miR-484 directly targeted Krüppel-like factor 4 (KLF4), a critical regulator of transcription factors necessary for cancer stem cell (CSC) development.^44^ Both miR-484 overexpression and KLF4 inhibition effectively re-sensitized resistant cells to tamoxifen by disrupting CSC formation.^44^ KLF4 was reported to orchestrate ERα signaling, and upregulation of KLF4 increased resistance to endocrine therapy.^86^

Overexpression of miR-375 re-sensitized tamoxifen-resistant breast cancer cells via direct targeting of metadherin (MTDH).^45^^,^^46^ The relevance of targeting MTDH was supported by the downregulation of miR-375 and the upregulation of MTDH in clinical samples of tamoxifen-treated patients and the correlation with poor survival.^45^^,^^46^ In an earlier study, MTDH was shown to mediate estrogen-independent growth and tamoxifen resistance by downregulating phosphatase and tensin homolog (PTEN).^87^ Moreover, it was shown that MTDH interacted with ERα in the nucleus upon stimulation with estrogen.^88^

miR-486 was involved in tamoxifen resistance through targeting high-mobility group AT-hook 1 (HMGA1).^47^ Overexpression of miR-486 and inhibition of HMGA1 restored tamoxifen sensitivity in resistant cells. Combining tamoxifen treatment with overexpression of miR-486 led to profound cell death and increased apoptosis.^47^ Interestingly, HMGA1 induced alternative splicing of Erα, resulting in increased levels of the ERα-46 isoform.^89^ Expression of this ERα isoform is reduced in tamoxifen-resistant breast cancer cells, inhibiting cell proliferation and expression of ERα-regulated target genes.^90^

It has become clear that the modulation of ER expression or functional activity is a resistance mechanism that is highly influenced by miRNAs at multiple levels. For some of the proven miRNA-target gene interactions, experimental work confirmed the relevance of the interaction with the proven target gene in relation to resistance, including let-7b and let-7i targeting the ESR1 transcript coding for the ERα36 protein, miR-484 targeting KLF4, and miR-486 targeting HMGA1.

## Signaling pathways activation

A second mechanism by which endocrine resistance can be achieved is via the deregulation of a variety of different signaling pathways.^11^ These include multiple growth factor pathways NOTCH and Wnt signaling. It has become clear that there is a bidirectional crosstalk between ER and these other signaling pathways. Twelve miRNAs were identified that regulate in total 13 genes that function within these pathways.

Elevated expression of ERBB2 (HER2 encoding gene) has been linked with tamoxifen resistance via ligand-independent activation of ERα.^91^ Expression of ERBB2 can be regulated by binding miR-26a/b and ELAV-like protein 1 (or HuR) to the 3′ UTR of ERBB2 transcripts. While binding of miR-26a/b leads to a decrease in ERBB2 expression, the binding of HuR stabilizes ERBB2 transcripts.^48^ Both forced expression of miR-26a/b and depletion of HuR resulted in the reversal of tamoxifen resistance.^48^

miR-186, a direct regulator of epiregulin (EREG), was shown to be downregulated in tamoxifen-resistant cells.^49^ EREG upregulation enhanced glycolysis via activation of the EGFR signaling pathway. Elevation of aerobic glycolysis has been associated with tamoxifen resistance in breast cancer.^92^ Treatment of mice bearing tamoxifen-resistant breast tumors with miR-186-3p decreased tumor growth and aerobic glycolysis.^49^

miR-21 inhibition enhances the sensitivity of breast cancer cells to tamoxifen and fulvestrant by inducing apoptosis.^50^ The effects observed upon the inhibition of miR-21 were caused by enhanced expression of its target PTEN and the subsequent inactivation of the PI3K-AKT-mTOR pathway.^50^ Loss of miR-489 was shown to promote tamoxifen resistance, while overexpression restored tamoxifen sensitivity. Overexpression of miR-489 inhibited its direct target MAPK14, which resulted in diminished phosphorylation of ERα. In addition, miR-489 was shown to target protein tyrosine phosphatase non-receptor type 11 (PTPN11), which affects the AKT/extracellular signal-regulated kinase pathway.^51^ Reduced levels of miR-214 were shown to contribute to tamoxifen and fulvestrant resistance by inducing autophagy.^52^ Most likely this was due to an upregulation of the miR-214 target gene uncoupling protein 2 (UCP2). Further investigations revealed a negative correlation between miR-214 and UCP2 in human breast cancer tissues.^52^ Overexpression of UCP2 was linked to activation of the PI3K-Akt-mTOR pathway, and this was linked to endocrine resistance in ER^+^ breast cancer.^52^ Decreased expression of miR-497 and miR-195 was linked with tamoxifen resistance by activating the PI3K-AKT pathway via direct targeting of RAF proto-oncogene serine/threonine-protein kinase (RAF1), AKT3, and MAP2K1 by these miRNAs.^53^ Enhanced expression of miR-497 and miR-195 and inhibition of the PI3K-AKT pathway both reversed tamoxifen sensitivity in tamoxifen-resistant cells.^53^ Expression of MIR497HG, the primary miRNA transcript for miR-497 and miR-195, was shown to be regulated by ERα in an estrogen-dependent way.^53^ miR-519a expression was high in tamoxifen-resistant cells compared to tamoxifen-sensitive cells, and inhibition of this miRNA restored tamoxifen sensitivity.^54^ PTEN, a suppressor of the PI3K-Akt pathway, was targeted by miR-519a.^54^ Overexpression of this target gene restored tamoxifen sensitivity in resistant cells. The relevance of these findings was supported by reduced levels of PTEN in breast cancer patients treated with tamoxifen and their association with poor survival.^54^

miR-190 enhanced tamoxifen sensitivity in tamoxifen-resistant breast cancer cells.^55^ This effect on tamoxifen sensitivity was shown to be caused by direct targeting of SRY-box transcription factor 9 (SOX9), thereby inhibiting the Wnt/β-catenin pathway.^55^ The inverse correlation of miR-190 and SOX9 in breast cancer tissue samples further substantiated the relevance of these findings. The effect of the miR-190-SOX9 interaction was also linked to zinc finger E-box binding homeobox 1 and ERα, which competitively regulated expression of miR-190. Interestingly, SOX9 was shown to promote the expression of FXYD domain containing ion transport regulator 3 (FXYD3), and FXYD3 was indispensable for the nuclear location of SOX9. FXYD3 interacted with Src and ERα, resulting in an activated complex that triggered Src to activate the non-genomic ERα signaling pathway and induce resistance to endocrine therapy.^93^

Elevated miR-155 expression was observed in tamoxifen-resistant cell lines and breast cancer patients. The relevance of miR-155 in tamoxifen resistance was shown by the overexpression and inhibition of miR-155, resulting in enhanced and decreased survival upon tamoxifen treatment, respectively.^56^ Suppressor of cytokine signaling 6 (SOCS6) was shown to be a direct target of miR-155, and overexpression of SOCS6 abrogated the miR-155-induced effects.^56^

Overexpressing miR-125b and miR-205 or silencing miR-424 induces resistance in AI-sensitive cells by regulating the AKT/mTOR downstream genes glycogen synthase kinase 3 beta (GSK3β) and ribosomal protein S6 kinase B1 (p70S6K or RPS6KB1).^57^ The inhibitory effects of all three miRNAs on these two proteins were shown by western blotting. Luciferase reporter assays confirmed direct targeting of GSK3β and p70S6K by miR-125b. Ectopic expression of miR-125b supported estrogen-independent growth of AI-sensitive cells. Moreover, elevated miR-125b was a prognostic marker for the outcome of breast cancer patients.^57^

Involvement of miRNAs in the regulation of signaling pathways activation was shown for several miRNAs targeting, for example, members of the PI3K-AKT-mTOR pathway. For miR-519 targeting PTEN, the phenotype was shown to be dependent on PTEN. Also, targeting of SOCS6 by miR-155 was shown to be associated with the observed phenotype for the response of endocrine therapy. In addition, miRNAs targeting ERBB2, EREG, or other pathways have been identified, but a causal role of the proven targets in the observed phenotypes was not shown.

### Cell-cycle modulation

Estrogen signaling induces, among others, cell-cycle progression, and treatment with endocrine therapies block cell-cycle progression at G1 phase of the cell cycle via ER-dependent mechanisms.^11^^,^^94^^,^^95^ Not surprisingly, endocrine-resistant breast cancers often exhibit alterations in cell-cycle regulators.^96^ A total of 10 miRNAs involved in resistance to endocrine therapy was identified, regulating 7 target genes related to cell-cycle regulation.

Overexpression of miR-221/222 was shown in breast cancer cells treated with tamoxifen or the tamoxifen metabolite 4-hydroxytamoxifen.^58^ Ectopic expression of this miRNA cluster induced tamoxifen resistance in MCF-7 cells by regulating the expression of CDK inhibitor 1B (CDKN1B).^58^^,^^59^ A second miRNA regulating the expression of CDKN1B is miR-575. Elevated miR-575 levels were observed in tamoxifen-resistant cells, and its depletion successfully overcame tamoxifen resistance in ERα^+^ breast cancer cells.^60^ Furthermore, overexpression of miR-575 could desensitize cells to tamoxifen, and this could be abolished by the simultaneous overexpression of cyclin D1, indicating the relevance of cyclin D1 for the miR-575-induced effects. Results were validated by the upregulation of miR-575 in ERα^+^ breast cancer tissue samples of patients with acquired resistance to tamoxifen.^60^ Expression of CCND1, the gene coding for cyclin D1, was in turn regulated by miR-497 and miR-195, and reduced expression of these miRNAs was linked to tamoxifen resistance.^53^ Another miRNA-target gene interaction related to cyclin D1 activity was miR-206 targeting the transcriptional coactivator WW domain binding protein 2 (WBP2). The relevance of this interaction was shown by either overexpression of miR-206 or inhibition of WBP2, which restored tamoxifen sensitivity in resistant cells. WBP2 influenced tamoxifen sensitivity by regulation expression of CDKN1A/p21, CCND1, and CDK4.^61^ Interestingly, WBP2 was also reported to enhance transactivation of ERα.^97^

Two negative regulators of the cell cycle, CDKN1A/p21 and retinoblastoma protein (RB1), were targeted by miR-519a.^54^ The relevance of these findings was supported by reduced levels of CDKN1A and RB1 in breast cancer patients treated with tamoxifen and their association with poor survival.^54^

The effect of miR-339 on tamoxifen resistance was shown to be achieved via its target gene CDK2. CDK2 expression was induced by the sponging of miR-339 by MAFG-AS1. The MAFG-AS1 gene contains an estrogen-responsive element, and its overexpression released CDK2 from miR-339-dependent regulation.^62^ The overexpression of miR-15a/16 resensitized tamoxifen-resistant cells via inhibition of its direct target cyclin E1 (CCNE1).^63^ CCNE1 functions as a regulatory subunit of CDK2, whose activity is required for cell-cycle G1/S transition.

In summary, miRNA-dependent resistance mechanisms involving the cell cycle mainly cluster around cyclin D1. The effects are achieved by either direct targeting of CCND1 or by targeting regulators of cyclin D1 expression. Direct proof of the relevance of the identified miRNA-target gene pair was shown for miR-575 targeting CCND1 and for miR-206 targeting WBP2.

### Other resistance mechanisms

Tamoxifen resistance in ERα^+^ breast cancer involves multiple cellular mechanisms that extend beyond the three mechanisms mentioned above. These mechanisms include regulatory feedback loops that influence cell growth and survival, the promotion of cell survival pathways, and the suppression of apoptosis.^11^ Additionally, resistance has been linked to alterations in pathways associated with the epithelial-to-mesenchymal transition (EMT), which has been associated with tamoxifen resistance in breast cancer.^98^ Changes in metabolic pathways, particularly those linked to glucose and serine synthesis, which provide energy and growth advantages, also contribute to resistance in cancer cells.^11^ Finally, modifications in cellular signaling promoting CSC phenotype and counteracting drug-induced cell death, contribute resistance.^11^ In total, 23 miRNAs were identified involved in such resistance mechanisms regulating 17 different target genes.

Both miR-26a knockdown and E2F transcription factor 7 (E2F7) overexpression induced tamoxifen resistance in ERα^+^ breast cancer cells.^64^ The relevance of these findings was supported by an inverse correlation between miR-26a and E2F7 in ERα^+^ breast cancer^64^ and by the correlation between E2F7 levels, the risk of relapse, and the poor prognosis in breast cancer patients treated with tamoxifen.^63^ Targeting of the transcription factor E2F7 by miR-26a resulted in repression of MYC. Lower MYC levels led to a decrease in miR-26a, revealing a feedback loop between miR-26a and E2F7 via MYC. E2F7 in turn can suppress miR-15a/16 expression, resulting in increased levels of the miR-15a/16 target B cell lymphoma 2 (BCL2).^63^ The overexpression of miR-15a/16 resensitized tamoxifen-resistant cells via inhibition of BCL2 resulting in apoptosis. In line with this, others showed that the inhibition of miR-15/16 in tamoxifen-sensitive cells induced the expression of BCL2 and promoted tamoxifen resistance.^65^ BCL2 was also shown to be regulated by miR-195 and miR-497. Decreased expression of these miRNAs was linked with tamoxifen resistance.^53^

Besides targeting MTDH, miR-375 re-sensitized tamoxifen-resistant breast cancer cells via direct targeting of homeobox B3 (HOXB3).^45^^,^^46^ Overexpression of HOXB3 induced a CSC phenotype, EMT, and tamoxifen resistance.^45^ An inverse relation has been reported between EMT and miR-200b/c levels in tamoxifen-resistant and tamoxifen-sensitive breast cancer cells. The miR-200b/c target gene MYB was upregulated in tamoxifen-resistant cells, and modulation of MYB levels influenced EMT and tamoxifen resistance.^66^ MYB is a downstream target of ERα signaling,^99^^,^^100^ and its expression was high in ER^+^ breast cancer samples. ER^+^ breast cancer cell lines were shown to be dependent on MYB expression, while ER^−^ breast cancer cell lines were not.^101^ Two additional miRNAs were implicated in regulating EMT as a resistance mechanism. For miR-19a-3p, the effect on EMT was linked to its target gene aromatase cytochrome P450 family 19 subfamily A member 1 (CYP19A1). *In vitro* data showed that upregulation of miR-19a in ERα^+^ breast cancer cells led to the induction of EMT and reduced sensitivity to AIs.^67^ For miR-27b, the link with EMT was shown via targeting HMGB3. Expression of miR-27b was decreased due to methylation of its promoter region in tamoxifen-resistant cells. Restoring expression of miR-27b made resistant cells more responsive to tamoxifen.^68^ Modulation of HMGB3 levels affected tamoxifen resistance, limited cell invasion, and counteracted EMT.^68^

Ectopic expression of miR-500a-3p sensitized ERα^−^ cells to tamoxifen.^31^ This was explained by inhibition of the expression of its direct target lymphocyte antigen 6 complex (LY6K). Decreased expression of LY6K induces decreased expression of its downstream target miR-192-5p, which regulates the expression of ERα (see above).^31^ Re-expression of ERα in ERα^−^ cells resulted in increased expression of miR-500a-3p and decreased expression of LY6K. This provides a regulatory loop involving two miRNAs, LY6K and ERα.

Overexpression of miR-449a re-sensitized cells to tamoxifen, while inhibition conferred resistance.^69^ This effect was shown to be dependent on the miR-449 target a disintegrin and metalloproteinase 22 (ADAM22). Silencing of ADAM22 could reverse the tamoxifen resistance that was induced by the inhibition of miR-449.^69^

Overexpression of miR-148a and miR-152 re-sensitized tamoxifen-resistant cells to tamoxifen.^70^ The effects were even more pronounced by the combined overexpression of miR-148a and miR-152. miR-148a and miR-152 directly targeted activated leukocyte cell adhesion molecule (ALCAM), and the potential role of ALCAM in resistance was validated by the overexpression of ALCAM.^70^ The clinical relevance of these findings was supported by increased ALCAM levels in breast tissues from tamoxifen non-responders compared to responders, as well as in resistant cell lines.^70^

miR-574 was identified through an miRNA overexpression screening approach in which dropout and retained miRNA constructs were identified in tamoxifen-treated compared to untreated cells. The effect of miR-574 on tamoxifen response was confirmed in independent experiments, and the mechanism was linked to its target gene, clathrin heavy chain (CLTC).^71^ Both overexpression of miR-574 and inhibition of CLTC restored sensitivity to tamoxifen.^71^ Moreover, decreased CLTC expression has been associated with improved survival among breast cancer patients treated with tamoxifen.^71^

Decreased levels of miR-145 and miR-424 and increased levels of miR-34b and miR-876 were shown in tamoxifen-resistant cells.^72^ Transient transfection of miR-145 and miR-424 restored sensitivity to endocrine therapy by targeting phosphoserine aminotransferase 1 (PSAT1), a key enzyme in the serine synthesis pathway previously linked to tamoxifen resistance.^72^ Inhibition of miR-34b and miR-876 resulted in the increased expression of phosphoglycerate dehydrogenase (PHGDH), another enzyme involved in the serine synthesis pathway, and this abolished tamoxifen resistance.^72^

miR-143 was downregulated and miR-155 was upregulated upon long-term estrogen deprivation, a model of AI resistance. Inhibition of miR-155 results in the upregulation of miR-143 and downregulation of miR-143 target gene hexokinase 2 (HK2), a gene important for glucose metabolism.^73^ It was postulated that the increase in miR-155 was responsible for the increased glycolytic metabolism as observed in a long-term estrogen-deprivation model.^73^

Tamoxifen treatment resulted in the downregulation of miR-575 expression, which is a downstream target of ERα. Upon binding to the miR-575 promoter, ERα activates transcription of miR-575. Overexpression of miR-575 decreased tamoxifen sensitivity by targeting breast cancer 1 (BRCA1), which resulted in the inhibition of ERα nuclear translocation.^60^ BRCA1 is a nuclear phosphoprotein that plays a critical role in DNA repair and maintaining genomic stability.^102^ In addition, BRCA1 can inhibit ERα signaling, which results in suppression of ERα downstream target genes.^103^ The relevance of miR-575 was supported by its upregulation in ERα breast cancer cells with acquired tamoxifen resistance.

Patients resistant to tamoxifen exhibited significant upregulation of miR-663b compared to tamoxifen responders. Inhibition of miR-663b in resistant cell lines blocked proliferation and induced apoptosis, resulting in enhanced sensitivity to tamoxifen.^74^ The miR-663b target gene tumor protein 73 (TP73) was proposed as a mediator of this effect, and its expression was downregulated in resistant cells.^74^

Using endocrine therapy for sensitive and resistant cell lines, a strong upregulation of miR-342 was observed in resistant cell lines.^75^ Restoring the levels of miR-342 in these cells resulted in re-sensitization of the cells to tamoxifen-induced apoptosis. The relevance of miR-342 was supported by decreased miR-342 expression in tamoxifen-non-responder patients.^75^ To identify potential targets of miR-342, a gene expression profiling study was conducted that revealed multiple targets, some of which (e.g., gem nuclear organelle-associated protein 4 [GEMIN4] and bone morphogenetic protein 7 [BMP7]) were validated as direct targets by reporter assays. GEMIN4 facilitates assembly of the spliceosomes and thereby acts as a regulator mRNA splicing. BMP7 is a ligand for various transforming growth factor β receptors and is involved in activation of the SMAD family of transcription factors. BMP7 was shown to antagonize the estrogen-induced breast cancer cell proliferation by inhibiting the MAPK pathway.^104^ However, the functional relevance of targeting these two genes for the observed effect of miR-342 on resistance has not been established.^75^

In this final group of miRNAs, the target genes are involved in a broad range of mechanisms to overcome sensitivity to anti-hormonal therapies. These findings further highlight the diverse influence of miRNAs on the success of breast cancer therapies. Among the proven targets, a functional role of the target genes was shown for miR-200b/c targeting MYB and affecting EMT, miR-27b targeting HMGB3, miR-449 targeting ADAM22, miR-148a and miR-152 both targeting ALCAM, and finally miR-574 targeting CLTC.

## miRNA-based strategies to overcome endocrine resistance

Besides miRNAs being able to target multiple genes, and individual genes being targeted by multiple miRNAs, a third point to consider is that miRNA-target gene interaction can vary between different cell types, with each having their own specific miRNA and gene expression profiles. Therefore, miRNA-based therapies should be designed carefully and used with caution. Extracellular vesicles and, in particular, exosomes are nanosized and represent a route by which bioactive cargoes such as lipids, proteins, and nucleic acids, including miRNAs, can enter a cell. It is therefore not surprising that natural but also synthesized nanoparticles have gained a particular interest for the delivery of miRNA-based therapies. They have distinct advantages, including a long circulating half-life, being well tolerated in humans, and good cell membrane-penetrating capacity. A main issue to consider is the specific targeting of the cell type of interest, which can be overcome through various targeting approaches.^105^ Some studies have demonstrated feasibility of miRNA-based nanoparticle approaches. For instance, patients with advanced solid tumors were treated with the liposomal miR-34a mimic MRX3488 in a phase 1 study. Despite some clinical benefits, the trial was closed early because of serious immune-mediated adverse events, including four deaths.^106^ Using EDV nanocells, miR-16 mimics impeded the growth of xenograft tumors *in vivo* in malignant pleural mesothelioma.^107^ Using the same delivery technique, miR-7 mimics were shown to inhibit the growth of adrenocortical carcinoma xenografts derived from both primary cells and cell lines.^108^ To further maximize the effect of miRNA-based therapies, one may consider co-delivering the miRNA-based therapy with other drugs. For example, co-loading anti-miR-21 and 4-hydroxytamoxifen into nanoparticles has shown enhanced anti-proliferative effects compared to individual treatments.^109^ This approach holds promise for synergistic therapy against breast cancer, enhancing the anti-proliferative effect by targeting both miR-21 and anti-hormonal drugs. Based on the miRNA-target gene interactions identified in this review, including several for which the target gene was shown to be critical for the observed effects, miRNAs could show promise in further investigations for their therapeutic potential.

## Concluding remarks and future perspectives

While treatment with tamoxifen, fulvestrant, and AIs are highly effective in adjuvant, curative, and palliative settings, 40% of patients eventually develop resistance over time.^110^ Despite the increased knowledge of the potential of miRNAs to modulate response to endocrine therapy, their use as therapeutic targets in the clinical setting is very limited. This review aimed to provide a comprehensive overview of miRNAs that can modulate response to anti-hormonal treatments. Overall, it is evident that miRNAs play crucial roles in regulating therapy sensitivity by targeting a diverse array of gene targets involved in various cellular processes, like ER modulation, activating signaling pathways and the cell cycle. This underscores the complexity of drug-resistance mechanisms in breast cancer, revealing a diverse array of miRNAs that target key genes and pathways involved in anti-hormonal resistance. These studies highlight the significance of miRNAs in driving therapeutic resistance and suggest the potential for miRNA-based interventions to restore treatment efficacy. Understanding the roles of these miRNAs in resistance mechanisms provides valuable insights into potential adaptive strategies utilized by cancer cells to evade therapeutic interventions. Our review highlights several promising candidates for miRNA-based therapies against ERα-resistant breast cancers. It is evident that multiple miRNAs can influence the response to anti-hormonal drugs and that a broad spectrum of mechanisms are involved based on the identified target genes.

## Methods

A PubMed literature search was conducted on September 17, 2024, to capture the relevant literature using MeSH terms to identify relevant articles on breast cancer, estrogen receptor, miRNAs, and drug resistance. The search strategy was conducted with the assistance of a professional librarian. The search query included MeSH terms (“Breast Neoplasms”[MeSH] OR “Breast”[MeSH] OR breast∗[tiab] OR mammar∗[tiab]) AND (“Neoplasms”[MeSH] OR Cancer∗[tiab] OR neoplasm∗[tiab] OR carcinoma∗[tiab] OR tumor∗[tiab] OR tumor∗[tiab] OR malignan∗[tiab] OR metastas∗[tiab]) AND (“Receptors, Estrogen”[MeSH] OR “er-positive”[tiab] OR “estrogen receptor∗”[tiab]) AND (“MicroRNAs”[MeSH] OR mirna∗[tiab] OR microrna∗[tiab] OR micro rna∗[tiab]) AND (“Drug Resistance”[MeSH] OR resistan∗[tiab] OR “Tamoxifen”[MeSH] OR “Fulvestrant”[MeSH] OR “Aromatase Inhibitors”[MeSH] OR Tamoxifen[tiab] OR Fulvestrant[tiab] OR “Aromatase Inhibitor∗”[tiab] OR ai[tiab]) NOT “Review” [Publication Type]. This search strategy was applied to find publications focused on the role of miRNAs in determining the response toward ER-targeted therapy and in inducing resistance in breast cancer patients. This revealed a total of 180 papers. Screening of titles and abstracts by two authors (Z.S.A.H. and A.v.d.B.) revealed 83 potentially relevant papers. The selected studies were reviewed for full text by three authors (Z.S.A.H., A.v.d.B., and J.K.), which yielded a total of 50 papers that met the inclusion criteria of showing a miRNA-dependent mechanism of resistance to any of the anti-hormonal drugs for this review. We focused on studies showing increased or decreased effectiveness of endocrine therapy upon modulation of expression of miRNAs. Experimentally validated target genes were listed when an interaction was proven by at least luciferase reporter assays or western blot. We grouped the miRNA-target gene interactions based on involvement of the target genes in currently known endocrine resistance mechanisms.

## Acknowledgments

We extend our deepest gratitude to Karin Sijtsma from Central Medical library/UMCG and her expertise in navigating PubMed using MeSH terms. Additionally, we acknowledge the Ministry of Higher Education, Research, and Innovation in Oman for their generous sponsorship of this work. The funder played no role in study design, data collection, analysis and interpretation of data, or the writing of this manuscript.

## Author contributions

Conceptualization, Z.S.A.H., A.v.d.B., and J.K. Methodology, Z.S.A.H., A.v.d.B., and J.K. Writing – original draft, Z.S.A.H., A.v.d.B., J.K. Writing – review & editing, A.v.d.B., J.K., B.v.d.V., and M.M. All authors have read and agreed to the published version of the manuscript.

## Declaration of interests

The authors declare no competing interests.

## Supplemental information

Document S1. Table S1

Document S2. Article plus supplemental information
