# Supplementary material for: miRNA-dependent resistance mechanisms to anti-hormonal therapies in estrogen receptor-positive breast cancer patients
Source: Mol Ther Oncol. 2025 Jan 28;33(1):200941. doi: 10.1016/j.omton.2025.200941 (PMC11969448; doi:10.1016/j.omton.2025.200941)
Supplement: Document S1. Table S1 [file mmc1.pdf]

OMTON, Volume *33*

Supplemental information

miRNA-dependent resistance mechanisms to anti-hormonal therapies in estrogen receptor-positive breast cancer patients

Zainab Salam Al Hashami, Bert van der Vegt, Marian J.E. Mourits, Joost Kluiver, and Anke van den Berg

Table S1. List of abbreviations

**ADAM22**	A Disintegrin And Metalloproteinase 22
**AKT**	AKT Serine/Threonine Kinase
**ALCAM**	Activated Leukocyte Cell Adhesion Molecule
**AI**	Aromatase Inhibitor
**BCL2**	B-cell lymphoma 2
**BMP7**	Bone Morphogenetic Protein 7
**BRCA1**	Breast Cancer 1
**CCND1**	Cyclin D1
**CCNE1**	Cyclin E1
**CDK2**	Cyclin Dependent Kinase 2
**CDK3**	Cyclin Dependent Kinase 3
**CDK4**	Cyclin Dependent Kinase 4
**CDKN1A**	Cyclin Dependent Kinase Inhibitor 1A
**CDKN1B**	Cyclin Dependent Kinase Inhibitor 1B
**CLTC**	Clathrin Heavy Chain
**CSC**	Cancer Stem Cell
**CNND1**	Cyclin D1
**CREB1**	cAMP-response element binding protein 1
**CTNNB1**	Catenin Beta 1 (part of Wnt signaling pathway)
**CUL4B**	Cullin 4B
**CYP19A1**	Cytochrome P450 Family 19 Subfamily A Member 1
**DGCR8**	DiGeorge Syndrome Critical Region Gene 8
**E2F7**	E2F Transcription Factor 7
**EGFR**	Epidermal Growth Factor Receptor
**EMT**	Epithelial-Mesenchymal Transition
**ERα**	Estrogen Receptor Alpha
**ERBB2**	Erb-B2 Receptor Tyrosine Kinase 2 (HER2)
**EREG**	Epiregulin
**ESR1**	Estrogen Receptor 1 Gene
**ESRRA**	Estrogen Related Receptor Alpha
**FOXP1**	Forkhead Box P1
**FXYD3**	FXYD Domain Containing Ion Transport Regulator 3
**GEMIN4**	Gem Nuclear Organelle Associated Protein 4
**GSK3B**	Glycogen Synthase Kinase 3 Beta
**HDAC**	Histone Deacetylase
**HER2**	Human Epidermal Growth Factor Receptor 2
**HK2**	Hexokinase 2
**HMGA1**	High Mobility Group AT-Hook 1
**HMGB3**	High Mobility Group Box 3
**HOXB3**	Homeobox B3
**HuR**	ELAV-Like Protein 1 (ELAV1)
**KLF4**	Krüppel-like factor 4

**LY6K**	Lymphocyte Antigen 6 Complex
**MAPK**	Mitogen-Activated Protein Kinase
**MIRNA**	MicroRNA
**MTDH**	Metadherin
**MTOR**	Mechanistic Target Of Rapamycin Kinase
**MYC**	Myc Proto-Oncogene
**NCOA1**	Nuclear Receptor Coactivator 1
**NR5A2**	Nuclear Receptor Subfamily 5 Group A Member 2
**P70S6K**	Ribosomal Protein S6 Kinase B1 (RPS6KB1)
**PHGDH**	Phosphoglycerate Dehydrogenase
**PI3K**	Phosphoinositide 3-Kinase
**PPP1R1B**	Protein Phosphatase 1 Regulatory Inhibitor Subunit 1B
**PTPN11**	Protein Tyrosine Phosphatase Non-Receptor Type 11
**PSAT1**	Phosphoserine Aminotransferase 1
**PTEN**	Phosphatase and Tensin Homolog
**RAF1**	RAF Proto-Oncogene Serine/Threonine-Protein Kinase
**SERM**	Selective Estrogen Receptor Modulator
**SERD**	Selective Estrogen Receptor Degrader
**SOCS6**	Suppressor of Cytokine Signaling 6
**SOX9**	SRY-Box Transcription Factor 9
**TGFB**	Transforming Growth Factor Beta
**TIMP3**	Tissue Inhibitor of Metalloproteinases 3
**TP73**	Tumor Protein 73
**UCP2**	Uncoupling Protein 2
**WBP2**	WW domain binding protein 2
**XPO5**	Exportin 5
**ZBTB10**	Zinc Finger And BTB Domain Containing 10
**ZEB1**	Zinc Finger E-Box Binding Homeobox 1
